# Cardiotoxicity of Immune Checkpoint Inhibitors with Anthracycline Chemotherapy in Triple-Negative Breast Cancer

**DOI:** 10.3390/curroncol33100574

**Published:** 2026-09-24

**Authors:** Nolan Holley, Syed Ahmad, Gary Monroe, Sonya Brooks, Pranathi Tella, Sobha Kurian, Brijesh Patel

**Affiliations:** 1 Department of Medicine, West Virginia University, Morgantown, WV 26506, USA; nolan.holley@osumc.edu (N.H.); gary.monroe2@hsc.wvu.edu (G.M.); pranathi.tella@hcahealthcare.com (P.T.); 2 Department of Oncology, West Virginia University, Morgantown, WV 26506, USA; 3 Department of Cardiology, Indiana University, Indianapolis, IN 46202, USA

**Keywords:** immunotherapy, anthracycline, triple-negative breast cancer, cardiotoxicity, cardiooncology, pembrolizumab

## Abstract

**Background/Aim**: The addition of an immune-checkpoint inhibitor (ICI) to chemotherapy improves outcomes in both early-stage and metastatic triple-negative breast cancer (TNBC). However, ICIs are associated with immune-related adverse events (irAEs), such as cardiotoxicity, raising concerns for patients receiving both anthracyclines and ICIs. We sought to determine if combining anthracycline and ICI in TNBC is associated with greater cardiotoxicity than anthracycline alone. **Methods**: A retrospective cohort study was conducted at a large academic center. Two cohorts were analyzed: anthracycline plus ICI and anthracycline alone. Demographics, comorbidities, medications, cardiac imaging, and outcomes were reviewed. Time to adverse cardiac events were compared. **Results**: A total of 382 patients were included: 297 received anthracyclines without ICI, and 85 received both. There were no major differences in baseline characteristics. The rate of acute cardiac events was 3.4% in the anthracycline-alone cohort and 3.5% in the anthracycline-plus-ICI cohort (*p* > 0.99) over a median of 1232 days of follow up (median of 1336 days in the anthracycline only group; median of 704 days in the ICI group). No significant differences in acute heart failure, acute coronary syndrome, myocarditis, or pericardial effusion were observed. Kaplan–Meier analysis showed no significant difference in cardiac event-free survival (*p* = 0.60). Furthermore, there was no significant difference in time to cardiac events between the two groups following competing risk analysis when accounting for both prior coronary artery disease (HR = 1.32, 95% CI: [0.42, 4.14], *p* = 0.64) and age (HR = 1.64, 95% CI: [0.52, 5.19], *p* = 0.40). **Conclusions**: This single-institution analysis did not identify a significant increase in cardiotoxicity with the addition of an ICI to anthracycline therapy in patients with TNBC. Active surveillance for cardiotoxicities should continue in patients receiving ICI in addition to anthracycline therapy.

## Simple Summary

The addition of immune checkpoint inhibitors (ICIs) to chemotherapy improves outcomes in triple-negative breast cancer (TNBC), but concerns remain about cardiotoxicity when combined with anthracyclines. We conducted a retrospective cohort study comparing patients treated with anthracyclines alone (*n* = 297) versus anthracyclines plus ICIs (*n* = 85). Baseline characteristics were similar between groups. Acute cardiac events occurred in 4.0% of the anthracycline-alone group and 4.7% of the combination group (*p* = 0.762), with no significant differences in heart failure, acute coronary syndrome, myocarditis, or pericardial effusion. Kaplan–Meier and competing risk analyses showed no significant differences in cardiac event-free survival or time to cardiac events after adjusting for age and prior coronary artery disease. These findings suggest that adding ICIs to anthracycline therapy does not significantly increase cardiotoxicity in patients with TNBC.

## 1. Introduction

Triple-negative breast cancer (TNBC) is an aggressive malignancy that necessitates neoadjuvant chemotherapy prior to surgical intervention when in its early stages (stages II–III). The standard-of-care chemotherapy regimen for early-stage high-risk TNBC consists of paclitaxel and carboplatin followed by or preceded by anthracycline (doxorubicin or epirubicin) and cyclophosphamide [1]. Initial regimens favored a dose-dense regimen (i.e., dosing every two weeks to prevent tumor cell growth) but have since favored dosing every three weeks with similar outcomes and improved tolerability [1]. In the setting of metastatic TNBC, anthracyclines also serve a pivotal role in management [2,3]. Despite these interventions, recurrence of disease and high rates of mortality remain an ongoing clinical challenge. Furthermore, anthracycline-based chemotherapy regimens are associated with substantial cardiotoxicity that require diligent monitoring of cardiac function and close clinical observation.

In a major alteration to the former standard-of-care, the KEYNOTE-522 trial showed significantly improved pathological complete response (pCR) at time of surgery, improved rates of event-free survival (EFS), and improved overall survival (OS) for patients with high-risk early-stage TNBC receiving an immune checkpoint inhibitor (pembrolizumab) with chemotherapy compared to those receiving chemotherapy alone, regardless of PD-L1 expression status [4,5,6]. This contributes to a growing body of evidence showing the utility of ICIs in treatment of TNBC. The American Society of Clinical Oncology recognizes the benefit of pembrolizumab in combination with chemotherapy in management of TNBC, recommending its use for previously untreated PD-L1 positive metastatic TNBC [3]. Atezolizumab, an alternative ICI, has seen decreased use after the IMpassion131 study [7,8]; thus, pembrolizumab remains the prominent ICI in TNBC management.

Cardiotoxicity is among the most worrisome of the immune-related adverse events (irAEs), most often manifesting as myocarditis, aberrant tachyarrhythmia, left ventricular dysfunction, and pericarditis [9]. Anthracycline use is proposed to cause direction oxidative stress and topoisomerase II dysfunction, while ICI use leads to indirect immune related cardiac injury. Given the baseline risk of cardiac dysfunction with anthracycline-based chemotherapy for high-risk early-stage TNBC, the addition of an ICI such as pembrolizumab raises concern for a theoretical increase in cardiovascular adverse events (cvAEs). In the KEYNOTE-522 trial, improved overall survival was seen with ICI therapy, but the reporting of cardiotoxicity profile was limited. Hence, we conducted a retrospective analysis to elucidate the burden of relevant cardiotoxicity at our academic medical institution.

## 2. Methods

### 2.1. Eligible Patients

This Institutional Review Board approved retrospective cohort study was conducted at the West Virginia University Hospital system. Patients aged ≥18 years with TNBC (April 2010 to September 2023) were identified using ICD-10 codes. Patients with <1 year of follow-up, except in cases of mortality, were excluded (Figure 1). Two cohorts were created: anthracycline-based chemotherapy alone (AC) or with ICI (AC + ICI).

### 2.2. Chemotherapy Regimen Details and Cardiotoxicity Surveillance

Demographics, comorbidities, relevant medications, and adverse cardiovascular events were collected from the patient electronic medical record. Patients were treated by their oncology teams in accordance with NCCN guidelines and patient specific-characteristics. Prior to KEYNOTE-522, the regimen of doxorubicin 60 mg/m^2^ and cyclophosphamide 600 mg/m^2^ for four cycles, and then paclitaxel 175 mg/m^2^ with a carboplatin agent, was the standard of care regimen [10]. After KEYNOTE-522, the use of pembrolizumab was incorporated into the treatment regimen.

Cardiotoxicity surveillance was conducted as standard for anthracycline exposure with determination of a pre-exposure echocardiogram, with possible repeat testing at one year post treatment, with earlier echocardiography if needed. Immune checkpoint inhibitor surveillance was conducted with symptom-driven biomarker and electrocardiogram monitoring.

### 2.3. Outcomes of Interest

Cardiac adverse events of interest included acute hypotension, acute heart failure, acute coronary syndrome, myocarditis, arrhythmia requiring cardioversion, pericardial effusion, and cardiac death. Acute hypotension was defined as new-onset hypotension acutely after administration of the chemo-immunotherapy medication(s) requiring hospitalization for management/evaluation; acute heart failure was defined as signs/symptoms of heart failure after chemo-immunotherapy administration (not required to be within 24 h of medication administration); and acute coronary syndrome was defined as troponin and/or electrocardiogram changes in association with classic symptoms of acute coronary syndrome as commonly clinically diagnosed. We also analyzed additional irAEs, including pneumonitis, colitis, dermatitis, and hepatitis. All irAEs were defined according to standard clinical definitions; myocarditis was diagnosed with typical clinical symptoms and laboratory signs (troponin elevation) and with acute coronary syndrome ruled out. Time to cardiac event, time to cardiac death, and time to all-cause mortality were compared among cohorts.

### 2.4. Statistical Analysis

Continuous data are shown as the median with associated interquartile range (IQR), while categorical data are shown as number with prevalence. Continuous variables were compared with the student’s *t*-test for means or Wilcoxon test for medians, and categorical variables were compared with Fisher’s exact test. Survival analysis was performed using Cox proportional hazards modeling to predict outcomes. Stepwise forward regression was used to determine the variables of interest to include in the Cox models. To prevent overfitting of the model and prevent generation of non-meaningful model results, the stepwise regression analysis was limited to selection of two variables given the number of outcome events identified in the cohort. Fine-Gray analysis was used to account for non-cardiovascular death as a competing risk when predicting outcomes of cardiac events or cardiovascular death. Statistical analysis was performed in RStudio version 4.5.1 using publicly available packages. *p*-values less than 0.05 were considered statistically significant.

## 3. Results

### 3.1. Baseline Characteristics

Among 382 total patients included in the study, 297 patients were in the AC group, and 85 patients in the AC + ICI group. The median ages of the AC and AC + ICI groups were 58.8 years (Q1–Q3 49.1–67.4) and 55.1 years (Q1–Q3 45.1–64.7), respectively. There was no statistically significant difference in age, BMI, cardiovascular medication use, diabetes, hypertension, coronary artery disease (CAD), PAD, CVA, heart failure, arrhythmias, or CKD between the two groups (*p* > 0.05, Table 1).

In the AC group, the distribution of stages 1, 2, 3 and 4 (metastatic) TNBC is as follows: *n* = 58, 134, 91, 14. In the AC + ICI group, the distribution of stages 1, 2, 3 and 4 (metastatic) TNBC is as follows: *n* = 7, 44, 33, 1. Cumulative anthracycline exposure for the entire study cohort was 240 mg/m^2^ (Q1–Q3 240–240) and did not significantly differ between patients treated with or without ICI (*p* = 0.18). The median cumulative ICI dose was 2400 mg (Q1–Q3 1400–3400) for patients on ICI. Patients who received ICI after March 2020 (after KEYNOTE-522, *n* = 69) had a higher cumulative ICI dose of 3000 mg (Q1–Q3 1770–3400), compared to those initiated prior to March 2020 (median dose 800 mg (Q1–Q3 800–5850), *p* = 0.014).

### 3.2. Adverse Events

Acute adverse cardiac events occurred in twelve patients (3.4%) receiving AC and in four patients (3.5%) receiving AC + ICI (*p* > 0.99). For patients treated with AC, three (1.0%) had hypotension of cardiac etiology requiring hospitalization, six (2.0%) had acute heart failure, three (1.0%) had acute coronary syndrome, and three (1.0%) had arrhythmia requiring cardioversion. For patients treated with AC + ICI, one (1.2%) had hypotension of cardiac etiology requiring hospitalization, one (1.2%) had acute heart failure, two (2.4%) had acute coronary syndrome, and one (1.2%) had arrhythmia requiring cardioversion. There were four cardiovascular deaths (1.3%) in the AC cohort and one cardiovascular death (1.2%) in the AC + ICI cohort. There were no differences in occurrence of myocarditis, and no patient was diagnosed with pericarditis (Table 2).

There were significantly more cases of immune-related colitis (*p* = 0.002) and hepatitis (*p* = 0.011) in patients receiving AC + ICI. There were no significant differences in dermatitis (*p* = 0.13). There were no patients in either cohort with nephritis. The incidence of all-cause mortality (*p* = 0.85), death due to malignancy (*p* = 0.16) and malignancy treatment (*p* = 0.40) was similar between groups. Among AC + ICI patients, other causes of death included pneumonia, septic shock, failure to thrive, or unknown (*n* = 3).

### 3.3. Survival Analysis

There were no significant differences between time to cardiac events or cardiac death (*p* = 0.60) between the groups (Figure 2). The median time of follow up was 1232 days (Q1–Q3 741–1272). The median time to a cardiac event was 139 days (Q1–Q3 83–355) and to cardiac death was 244 days (Q1–Q3 90–462) for the AC group. The median time to a cardiac event was 197 days (Q1–Q3 190–509) and to cardiac death was 353 days (Q1–Q3 195–616) for the AC + ICI group. Furthermore, we identified no significant differences in time to cardiac event (*p* = 0.86), or time to cardiac event or all-cause mortality between groups (*p* = 0.49). Stepwise regression identified the presence of CAD and age as the top predictors of adverse cardiac outcomes. A Cox hazards model with CAD and regimen choice showed no statistical increase in the hazard of cardiac events or cardiac death with use of ICI (*p* = 0.64). A separate model with age and regimen choice also did not show an increased hazard for events from regimen choice (*p* = 0.40). As mentioned in the methods, only two covariates (age and CAD) were utilized in the multivariable models to prevent overfitting of the models. Other comorbidities did not significantly predict the risk of adverse cardiotoxicities. Fine-Gray analysis investigated the effect of death from non-cardiovascular causes as a competing risk for cardiac events and cardiac death; neither the model with CAD and regimen choice (regimen choice HR 1.30, *p* = 0.65) nor the model with age and regimen choice (HR 1.57, *p* = 0.45) showed an increase in events with AC + ICI (Table 3).

## 4. Discussion

The results of this study suggest that there are no significant differences in cardiac events or cardiac event-free survival between patients with TNBC treated with anthracycline-based chemotherapy with or without an immune checkpoint inhibitor. While each agent of this chemotherapy regimen has associated toxicities, anthracyclines such as doxorubicin are of the greatest concern to cardio-oncologists. Patients are closely monitored with follow-up for elucidation of symptoms such as dyspnea on exertion and lower extremity edema as these symptoms could be indicative of a reduction in left ventricular ejection fraction or global longitudinal strain. Professional organizations differ significantly in their recommendations for cardiac monitoring before, during, and after anthracycline-based chemotherapy. However, guidelines generally agree that the use of cardiac imaging, with or without biomarker monitoring, is essential for this patient population [11,12,13,14,15,16,17].

Pembrolizumab has a central role in the management of metastatic TNBC and high-risk early-stage TNBC and has been shown to significantly improve rates of pCR prior to surgery, EFS, and OS [4,5,6]. Since adjuvant pembrolizumab is a category 2A recommendation, many clinicians consider the possibility of the development of irAEs when engaging in a risk–benefit discussion with patients regarding the choice for use of adjuvant pembrolizumab. We show that there are no increases in cvAEs when an ICI is added to standard-dose anthracycline chemotherapy in this single-institution study. Patient selection for anthracycline therapy should continue to be carefully conducted given the known toxicities with anthracycline therapy, and optimization of pre-existing heart failure should be considered prior to therapy initiation.

Immunotherapy agents used in TNBC prevent the binding of PD-L1 presented by the tumor to the PD-1 receptor of T-cells. This disrupts the mechanism of immune evasion for the tumor cell and ultimately allows for the detection and destruction of tumor cells. Immune-related adverse events are hypothesized to be caused by an aberrant loss of immune evasion of innocuous tissues. Anthracycline-related toxicity, conversely, is suspected to be due to oxidative stress, free radicals exposure, and topoisomerase II inhibition, leading to direct cardiotoxic effects. The potential synergistic effect of anthracycline plus ICI therapy leading to myocarditis is most concerning for patients with early-stage TNBC as they may have longer survival compared to those with metastatic disease, increasing the time during which cardiotoxicity may develop. This effect, however, was not seen, with no difference recorded in myocarditis events between the groups (*p* > 0.99).

Anthracyclines and ICIs confer risk for cardiovascular toxicity through different independent mechanisms. For this reason, many oncologists, cardiologists, and cardio-oncologists have had reservations with regards to the cardiac safety of the anthracycline plus ICI regimen. We present this study with important findings that are reassuring for clinicians and patients using a new standard-of-care regimen for TNBC. This study suggests that the use of chemoimmunotherapy is not associated with a significant increase in risk of cardiac adverse events, time to cardiac event, or time to cardiac death. Thus, these findings support the use of chemoimmunotherapy for TNBC [18]. Furthermore, these findings suggest that the current guidelines for cardiac surveillance of patients on anthracycline chemotherapy, which recommend baseline cardiac risk assessment, echocardiographic monitoring, cardio-oncology referral, and symptom monitoring, are likely sufficient for those on chemoimmunotherapy as well.

### Limitations

This study is limited by the sample size given the nature of a single-institution retrospective analysis. There were less patients in the AC + ICI cohort given that the use of ICI in TNBC is a recent addition to standard-of-care chemotherapy. Furthermore, the incidence of cardiac events in both cohorts was low, allowing for the possibility of a type II error secondary to a small sample size. Due to the small number of events, multivariable models were limited to two predictor variables (the use of ICI with either age or CAD status); although more predictor variables could further identify risk for cardiac events, the main variable of interest in the study was ICI use, which did not show a statistically significant increase in risk for cardiac events in our model. Given the primary aim of this study was not to report a comprehensive oncologic profile of the patients, the pathological complete response and disease-free survival data were not reported. Given that this dataset represents real-world practice and is limited by its retrospective nature, it is possible that some patients’ adverse events were missed by the clinician overseeing the patient’s care. Future studies are warranted to expand on these findings in a larger multi-national cohort study with increased power for the further validity and reliability of these findings.

## 5. Conclusions

Treatment regimens with a high risk of cardiotoxicity in TNBC, such as anthracycline plus ICI use, require surveillance for the development of possible cardiotoxicities. In this study, the addition of an ICI to anthracycline-based chemotherapy was not associated with increased cardiovascular adverse events. These findings support the cardiac safety of current chemotherapy regimens and suggest that standard cardiovascular monitoring guidelines for anthracycline-based chemotherapy are likely sufficient for patients receiving concurrent ICI therapy.

## Figures and Tables

**Figure 1:**
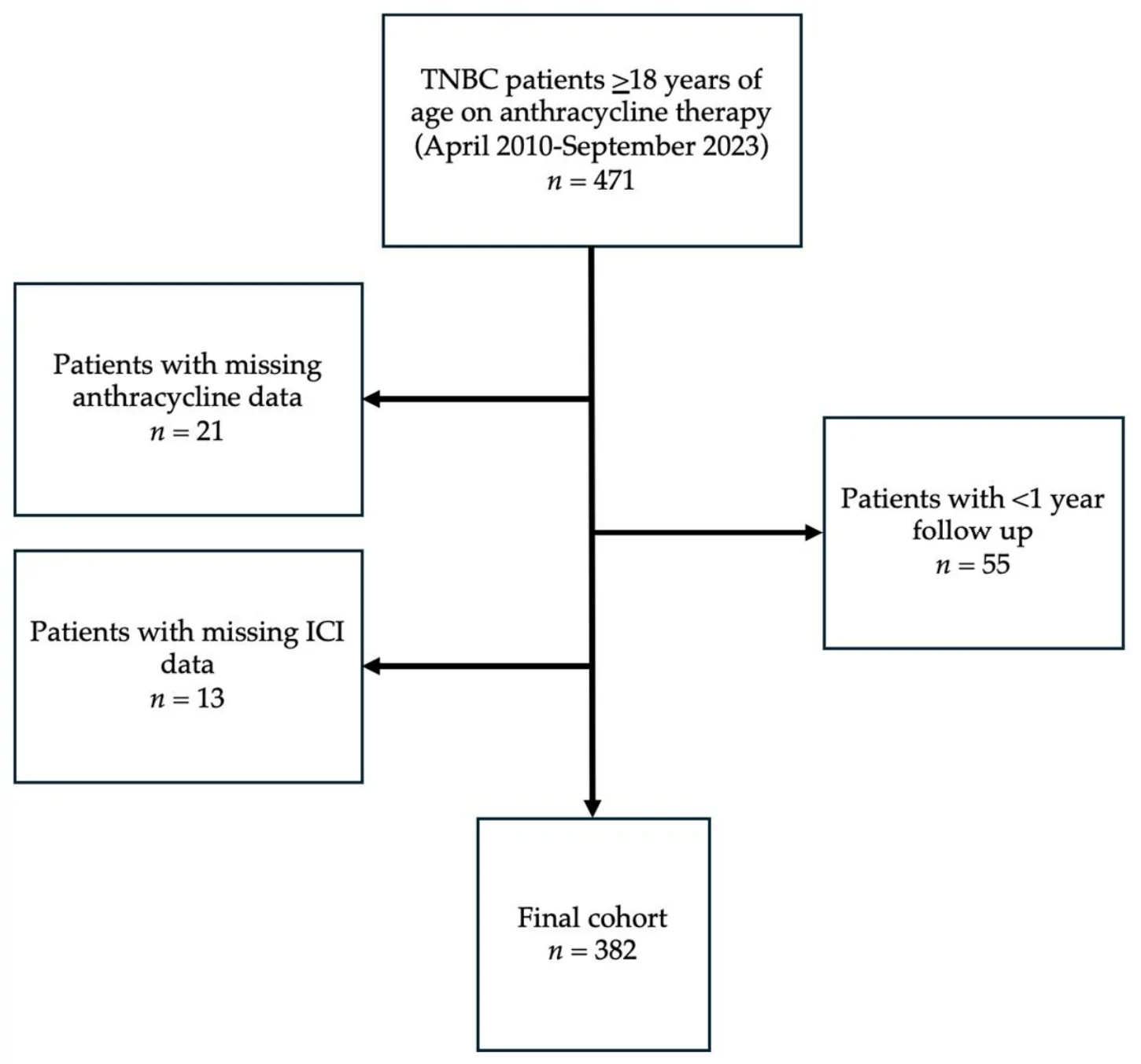
Patient selection flow diagram.

**Figure 2:**
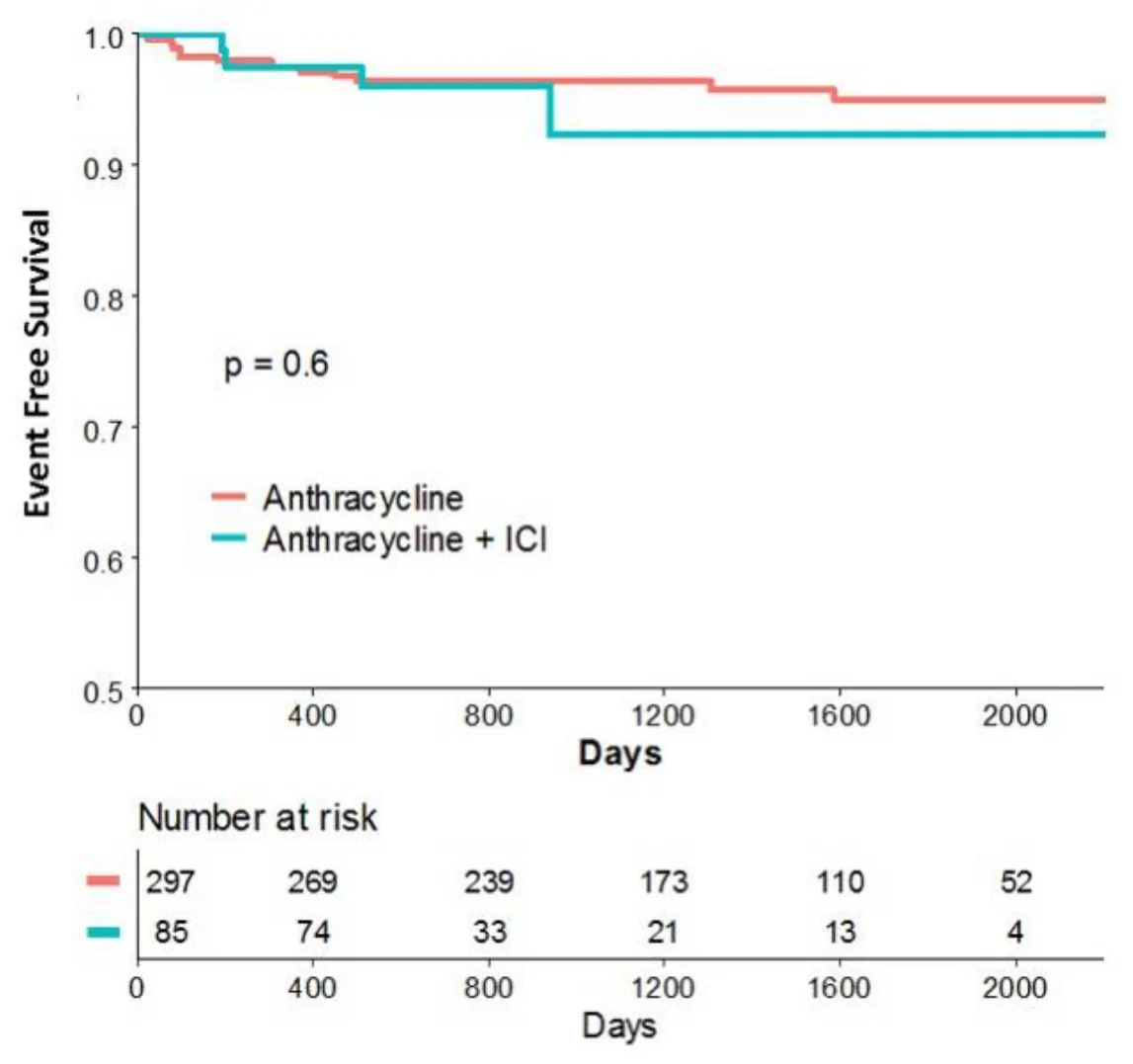
Kaplan–Meier estimate for cardiac event-free survival. Patients in the AC cohort had a median time to cardiac event of 139 days and median time to cardiac death of 244 days, while patients in the AC + ICI cohort had a median time to cardiac event of 197 days and median time to cardiac death of 353 days (*p* = 0.60).

**Table 1:** Baseline characteristics.

	All Patients	Anthracycline	Anthracycline + Immunotherapy	*p*-Value
	(*n* = 382)	(*n* = 297)	(*n* = 85)	
Demographics				
Female	376 (98.4%)	291 (98.0%)	85 (100%)	0.35
Age, median (IQR)	57.1 (48.3, 66.3)	58.8 (49.1, 67.4)	55.1 (45.1, 64.7)	0.075
BMI, median (IQR)	30.7 (26.1, 35.9)	30.7 (26.1, 35.9)	31.5 (26.9, 35.4)	0.94
Medications				
Aspirin	54 (14.1%)	41 (13.8%)	13 (15.3%)	0.73
ACEi/ARB	105 (27.5%)	82 (27.6%)	23 (27.1%)	>0.99
Beta-blocker	49 (12.8%)	39 (13.1%)	10 (11.8%)	0.86
Loop diuretic	26 (8.7%)	20 (6.7%)	6 (7.1%)	0.81
Thiazide diuretic	25 (6.5%)	20 (6.7%)	5 (5.9%)	>0.99
MRA	10 (2.6%)	10 (3.4%)	0 (0.0%)	0.13
SGLT2i	31 (8.1%)	26 (8.8%)	5 (5.9%)	0.50
Statin	91 (23.8%)	70 (23.6%)	21 (24.7%)	0.89
Cumulative anthracycline exposure (mg/m^2^)	240 (240, 240)	240 (240, 240)	240 (240, 240)	>0.99
Comorbidities				
DMNID	47 (12.3%)	33 (11.1%)	14 (16.5%)	0.19
IDDM	19 (5.0%)	15 (5.1%)	4 (4.7%)	>0.99
HTN	160 (41.9%)	125 (42.1%)	35 (41.2%)	0.90
HLD	123 (32.2%)	88 (29.6%)	35 (41.2%)	0.049
Obesity	200 (52.4)%	157 (52.9%)	43 (50.6%)	0.71
Prior coronary artery intervention	7 (1.8%)	5 (1.7%)	2 (2.4%)	0.65
No prior coronary interventions	13 (3.4%)	10 (3.4%)	3 (3.5%)	>0.99
PAD	1 (0.3%)	1 (0.3%)	0 (0%)	>0.99
CVA	6 (1.6%)	5 (1.7%)	1 (1.2%)	>0.99
HFrEF	3 (0.8%)	3 (1.0%)	0 (0%)	>0.99
HFpEF	11 (2.9%)	11 (3.7%)	0 (0%)	0.13
Atrial fibrillation	9 (2.4%)	8 (2.7%)	1 (1.2%)	0.69
CKD	14 (3.7%)	12 (4.0%)	2 (2.4%)	0.74

ACEi—angiotensin converting enzyme inhibitor, ARB—angiotensin II receptor blocker, BMI—body mass index, CKD—chronic kidney disease, CVA—cardiovascular accident, DMNID—diabetes mellitus not insulin-dependent, HFrEF—heart failure with reduced ejection fraction, HFpEF—heart failure with preserved ejection fraction, HLD—hyperlipidemia, HTN—hypertension, IDDM—insulin-dependent diabetes mellitus, IQR—interquartile range, MRA—mineralocorticoid receptor antagonist, and PAD—peripheral artery disease.

**Table 2:** Events summary.

	All Patients	Anthracycline	Anthracycline + ICI	*p*-Value
	(*n* = 382)	(*n* = 297)	(*n* = 85)	
**Acute cardiac event**	13 (3.4%)	10 (3.4%)	3 (3.5%)	>0.99
**Hypotension**	4 (1.0%)	3 (1.0%)	1 (1.2%)	>0.99
**Acute CHF**	7 (1.8%)	6 (2.0%)	1 (1.2%)	>0.99
**ACS**	5 (1.3%)	3 (1.0%)	2 (2.4%)	0.31
**Arrhythmia requiring cardioversion**	4 (1.0%)	3 (1.0%)	1 (1.2%)	>0.99
**Myocarditis**	4 (1.0%)	3 (1.0%)	1 (1.2%)	>0.99
**Pneumonitis**	1 (0.3%)	1 (0.3%)	0 (0%)	>0.99
**Colitis**	4 (1.0%)	0 (0%)	4 (4.7%)	0.002
**Dermatitis**	3 (0.8%)	1 (0.3%)	2 (2.4%)	0.13
**Hepatitis**	3 (0.8%)	0 (0%)	3 (3.5%)	0.011
**Pericardial effusion**	1 (0.3%)	0 (0%)	1 (1.2%)	0.22
**All-cause mortality**	47 (12.3%)	36 (12.1%)	11 (12.9%)	0.85
**Cardiovascular death**	5 (1.3%)	4 (1.3%)	1 (1.2%)	>0.99
**Death due to malignancy**	29 (7.6%)	26 (8.8%)	3 (3.5%)	0.16
**Death due to malignancy treatment**	2 (0.5%)	1 (0.3%)	1 (1.2%)	0.4
**Other**	11 (2.9%)	5 (1.7%)	6 (7.1%)	0.04
**Cardiac event or cardiac death**	16 (4.2%)	12 (4.0%)	4 (4.7%)	0.76

Acute cardiac events include hypotension of cardiac etiology requiring hospitalization; acute congestive heart failure (CHF); acute coronary syndrome (ACS); and arrhythmia requiring cardioversion. The were no significant differences in acute cardiac events (*p* = 0.76) or other irAEs apart from colitis (*p* = 0.002). Overall, there were no significant differences in cardiac events or cardiac death.

**Table 3:** Univariable, multivariable, and Fine-Gray competing-risks regression analyses.

Model 1	Univariable HR	Multivariable HR	Fine-Gray Analysis HR
AC + ICI	1.35 (0.43–4.24, *p* = 0.60)	1.32 (0.42–4.14, *p* = 0.64)	1.30 (0.41–4.12, *p* = 0.65)
CAD	4.48 (1.28–15.72, *p* = 0.019)	4.44 (1.26–15.59, *p* = 0.020)	4.55 (1.34–15.43, *p* = 0.015)
**Model 2**			
AC + ICI	1.35 (0.43–4.24, *p* = 0.60)	1.64 (0.52–5.19, *p* = 0.40)	1.57 (0.49–5.05, *p* = 0.45)
Age	1.07 (1.02–1.12, *p* = 0.007)	1.07 (1.02–1.12, *p* = 0.006)	1.07 (1.03–1.11, *p* = 0.001)

Results of stepwise forward-selection modeling for predictors of adverse cardiac outcomes following anthracycline therapy with or without an immune checkpoint inhibitor (ICI) are shown. Univariable and multivariable analyses, as well as Fine-Gray competing-risks regression for noncardiac death, are displayed. Estimates are presented as hazard ratios (HR) with 95% confidence intervals. Coronary artery disease (CAD) and increasing age emerged as independent predictors of cardiac events.

## Data Availability

Data may be made available upon reasonable request.

## List of Abbreviations

AC	Anthracycline chemotherapy
CAD	Coronary artery disease
cvAE	Cardiovascular adverse event
EFS	Event-free survival
ICI	Immune checkpoint inhibitor
irAE	Immune-related adverse event
OS	Overall survival
pCR	Pathological complete response
TNBC	Triple-negative breast cancer
TTE	Transthoracic echocardiogram
